# IGSF1 Deficiency Results in Human and Murine Somatotrope Neurosecretory Hyperfunction

**DOI:** 10.1210/clinem/dgz093

**Published:** 2019-10-25

**Authors:** Sjoerd D Joustra, Ferdinand Roelfsema, A S Paul van Trotsenburg, Harald J Schneider, Robert P Kosilek, Herman M Kroon, John G Logan, Natalie C Butterfield, Xiang Zhou, Chirine Toufaily, Beata Bak, Marc-Olivier Turgeon, Emilie Brûlé, Frederik J Steyn, Mark Gurnell, Olympia Koulouri, Paul Le Tissier, Pierre Fontanaud, J H Duncan Bassett, Graham R Williams, Wilma Oostdijk, Jan M Wit, Alberto M Pereira, Nienke R Biermasz, Daniel J Bernard, Nadia Schoenmakers

**Affiliations:** 1 Department of Medicine, Division of Endocrinology, Leiden University Medical Center, Leiden, Netherlands; 2 Department of Pediatrics, Leiden University Medical Center, Leiden, Netherlands; 3 Emma Children’s Hospital, Amsterdam UMC, University of Amsterdam, Pediatric Endocrinology, Amsterdam, Netherlands; 4 Department of Endocrinology, Ludwig-Maximilians University, Munich, Germany; 5 Department of Radiology, Leiden University Medical Center, Leiden, Netherlands; 6 Molecular Endocrinology Laboratory, Department of Medicine, Imperial College London, London, UK; 7 Departments of Anatomy and Cell Biology & Pharmacology and Therapeutics, McGill University, Montreal, Quebec, Canada; 8 The University of Queensland Centre for Clinical Research, Brisbane, Australia; 9 University of Cambridge Metabolic Research Laboratories, Wellcome Trust-Medical Research Council Institute of Metabolic Science, Addenbrooke’s Hospital, Cambridge CB2 0QQ UK; 10 Centre for Integrative Physiology, University of Edinburgh, Edinburgh, UK; 11 CNRS, Institut de Génomique Fonctionnelle, INSERM, and Université de Montpellier, Montpellier, France

**Keywords:** pituitary, IGSF1, growth hormone, congenital hypopituitarism

## Abstract

**Context:**

The X-linked immunoglobulin superfamily, member 1 (*IGSF1*), gene is highly expressed in the hypothalamus and in pituitary cells of the POU1F1 lineage. Human loss-of-function mutations in *IGSF1* cause central hypothyroidism, hypoprolactinemia, and macroorchidism. Additionally, most affected adults exhibit higher than average IGF-1 levels and anecdotal reports describe acromegaloid features in older subjects. However, somatotrope function has not yet been formally evaluated in this condition.

**Objective:**

We aimed to evaluate the role of IGSF1 in human and murine somatotrope function.

**Patients, Design, and Setting:**

We evaluated 21 adult males harboring hemizygous IGSF1 loss-of-function mutations for features of GH excess, in an academic clinical setting.

**Main Outcome Measures:**

We compared biochemical and tissue markers of GH excess in patients and controls, including 24-hour GH profile studies in 7 patients. Parallel studies were undertaken in male *Igsf1*-deficient mice and wild-type littermates.

**Results:**

IGSF1-deficient adult male patients demonstrated acromegaloid facial features with increased head circumference as well as increased finger soft-tissue thickness. Median serum IGF-1 concentrations were elevated, and 24-hour GH profile studies confirmed 2- to 3-fold increased median basal, pulsatile, and total GH secretion. Male *Igsf1*-deficient mice also demonstrated features of GH excess with increased lean mass, organ size, and skeletal dimensions and elevated mean circulating IGF-1 and pituitary GH levels.

**Conclusions:**

We demonstrate somatotrope neurosecretory hyperfunction in IGSF1-deficient humans and mice. These observations define a hitherto uncharacterized role for IGSF1 in somatotropes and indicate that patients with *IGSF1* mutations should be evaluated for long-term consequences of increased GH exposure.

*IGSF1* encodes a transmembrane immunoglobulin superfamily glycoprotein that is highly expressed in the Rathke pouch (the developing pituitary primordium), adult anterior pituitary cells, and the hypothalamus. X-linked, loss-of-function mutations in *IGSF1* result in a variable spectrum of anterior pituitary dysfunction, which is most pronounced in affected males, resulting almost universally in mild to moderate congenital central hypothyroidism (CCeH). Additionally, affected boys generally exhibit discordant pubertal development with delayed testosterone rise and growth spurt, but normal or precocious onset of testicular growth and subsequent postpubertal macroorchidism. Hypoprolactinemia also affects 60% of cases (1–3). Heterozygous female mutation carriers may demonstrate no overt endocrinopathies; however, up to 20% heterozygotes also exhibit CCeH or hypoprolactinemia, and an association with delayed menarche has also been reported (3, 4).

IGSF1 undergoes cotranslational proteolysis such that only its carboxy-terminal domain (CTD) is trafficked to the plasma membrane (5). Disease-associated *IGSF1* mutations include entire gene deletions as well as missense, nonsense, and frameshift mutations principally in the part of the gene encoding the CTD. Intragenic mutations usually impair trafficking and membrane localization of the CTD when assessed by overexpression in heterologous cells (1, 3).

Although crucial for normal pituitary hormone production, the physiological and molecular role of IGSF1 remains to be elucidated. In this regard, 2 different *Igsf1*-deficient mouse models (*Igsf1*^∆exon1^ and *Igsf1*^∆312^) have yielded valuable functional insights. Males of both strains exhibit CCeH, decreased expression of thyrotropin-releasing hormone receptor 1 (*Trhr1*) mRNA in their pituitary, and impaired responsiveness to thyrotropin-releasing hormone (TRH). These results support a mechanistic role for impaired TRH action in the pathogenesis of CCeH (1, 6).

Somatotrope function has not yet been formally evaluated in IGSF1 deficiency; however, existing data suggest that there may be a complex perturbation of GH homeostasis. In humans harboring hemizygous *IGSF1* mutations, partial transient GH deficiency occurs in 16% of boys. However, in adulthood, IGF-1 levels usually rise above the mean and are anecdotally associated with acromegaloid facial features consistent with GH excess (3, 4). *Igsf1*-deficient male mice exhibit increased body mass, but body composition has not been evaluated to establish whether lean mass, organ size, and skeletal dimensions are also increased, as predicted for a GH-mediated effect (1). A lack of robust antihuman IGSF1 antibodies precludes characterization of IGSF1 expression in human pituitary; therefore, protein expression studies have been undertaken in rodents. Studies in mice and rats using a custom IGSF1 antibody detected IGSF1 expression in all cells of the POU1F1 lineage, including somatotropes, consistent with a role for IGSF1 in GH production (1, 7). These results are substantiated by recent single-cell RNA sequencing data from adult male mouse pituitaries (8).

We evaluated somatotrope function in male IGSF1-deficient humans and mice, profiling GH secretion, delineating GH expression in murine pituitary, and quantifying peripheral GH-mediated effects in both species. Our data demonstrate somatotrope neurosecretory hyperfunction in both species, with associated end-organ sequelae of GH excess.

## Materials and Methods

### Study approval

Investigations were undertaken with Research Ethics Committee approval (Leiden University Medical Center, P13.008) with prior written, informed consent and/or were clinically indicated. Mouse studies were regulated under the Animals (Scientific Procedures) Act 1986 Amendment Regulations 2012 following ethical review by the University of Cambridge Animal Welfare and Ethical Review Body (UK Home Office License no. 80/2098) or conducted in accordance with federal and institutional guidelines with approval of the Goodman Cancer Centre Facility Animal Care Committee, McGill University (protocol no. 5204).

### IGSF1-deficient patient cohort

Clinical studies were performed in the Leiden University Medical Center and Addenbrooke’s Hospital, Cambridge. General auxological and physiological features of GH hypersecretion have been evaluated in a previously published cohort of 69 male patients (3). All individuals harbored hemizygous *IGSF1* mutations defined as pathogenic on the basis of associated CCeH, phenotype-genotype segregation, and in silico and in vitro characterization (3). Treatment with levothyroxine was used in 89% of boys and 44% of adult males.

For the current study, 21 adult males from this cohort were recruited from the Netherlands and the United Kingdom for targeted evaluation of GH excess and its associated sequelae using clinical assessment and direct questioning. This cohort will be referred to henceforth as the targeted cohort; their characteristics are summarized in Table 1. All 21 patients had CCeH (16 were taking levothyroxine), 10 of 21 were prolactin deficient, 1 had received rhGH for GH deficiency, and most had exhibited delayed pubertal development, for which 1 still received testosterone replacement. Cortisol levels were normal and testosterone mildly decreased in 2 of 21 cases. Their ages ranged from 19 to 89 years (median age, 55.1 years; P10-P90, 21.8-85.2 years) and the age distribution was skewed toward the older part of the range (Shapiro-Wilk test *P *= 0.015).

**Table 1.** Indices of GH Action in Targeted Cohort of IGSF1-Deficient Male Patients

	Patients	Reference Data Source and Range	*P* Value
n	21		
Age (y)	55.1 (21.8–85.2)		
BMI (kg/m^2^)	27.4 (24.4–34.6)		
Classified as acromegalic (%)^a^	52.4	19.1^b^	0.024
Finger soft-tissue thickness (SDS)^c^	1.1 (-0.3 to 2.3)	(9)	0.001
Adult height SDS	-0.4 (-1.7 to 1.0)	(10, 11)	0.138
Head circumference (SDS)	1.3 (0.1–2.4)	(12)	<0.001
Fat mass index (SDS)^d^	-0.08 (-0.94 to 4.95)	(13)	0.175
Fat free mass index (SDS)^d^	0.40 (-2.42 to 1.92)	(13)	0.793
IGF-1 (SDS)	1.0 (-0.1 to 2.9)	*In-house (age-dependent)*	0.001
Free T4 (pmol/L)	14.3 (8.9–21.9)	12.0–22.0 pmol/L	
Prolactin (µg/L)	4.3 (1.4–15.4)	4.0–15.0 µg/L	
Testosterone (nmol/L)	14.3 (7.9–22.1)	8.0–31.0 nmol/L	
Cortisol (µmol/L)	0.355 (0.196–0.533)	0.100–0.600 µmol/L	
Testicular volume (SDS)^e^	2.9 (1.2–5.8)	(14)	<0.001

Data presented as number or median (P10-P90).

Abbreviations: BMI, body mass index; SDS, standard deviation score.

^a^Based on software analysis of facial photographs.

^b^Based on 18 in-house age- and BMI-matched controls.

^c^N = 20.

^d^Corrected for BMI and height, available n = 19 for fat mass index and n = 16 for fat-free mass index.

^e^Ultrasonographic volume of largest testis, n = 16.

Seven Dutch IGSF1-deficient adult males from this cohort consented to more detailed evaluation of 24-hour GH secretion patterns. These individuals were selected on the basis of their ability to attend Leiden University Medical Center for these studies and their willingness to undertake this intensive sampling regimen. These 7 adult male patients exhibited similar phenotypic characteristics and endocrinopathies (hypoprolactinemia, levels of IGF-1 and testosterone) to adult male patients in the targeted cohort of 21 males, and their characteristics are summarized in Table 2. In 1 (patient 7), a TRH test with measurement of GH was available, showing a paradoxical GH response. All patients had CCeH, 5 of 7 were on levothyroxine replacement, with mean FT4 11.3 ± 4.3 pmol/L. None had recently taken transmeridianal flights or carried out night shift work and all patients did not smoke. GH secretion patterns were compared with available data using 2 age- and body mass index (BMI)-matched healthy male control subjects per patient, sampled using the same protocol and exclusion criteria (15–17).

**Table 2.** GH Secretion in 7 IGSF1-Deficient Male Patients

Patient No.	1	2	3	4	5	6	7	Patients (n = 7)	Controls (n = 14)	*P* value
Age (y)	23.8	28.2	22.0	62.5	22.2	18.4	53.5	24 (20–56)	35 (26–61)	0.135
BMI (kg/m^2^)	24.8	29.5	25.9	34.4	27.1	29.4	27.3	27.3 (25.5–31.5)	24.5 (21.8–29.9)	0.263
IGF-1 (SDS)^a^	0.6	2.2	0.1	2.0	-1.2	0.2	2.7	0.9 ± 1.3	-0.4 ± 0.8	0.043
Basal GH secretion (µg/L/24 h)	3.50	4.58	1.82	2.74	2.11	22.38	3.50	3.50 (1.99–11.70)	1.05 (0.27–3.30)	0.014
Pulsatile GH secretion (µg/L/24 h)	52.59	29.84	34.18	17.17	47.69	100.54	21.35	34.18 (19.68–71.77)	18.99 (6.58–30.99)	0.009
Total GH secretion (µg/L/24 h)	56.09	34.41	36.00	19.91	49.78	122.92	24.85	36.00 (22.87–82.82)	20.83 (7.55–32.89)	0.006
ApEn (dimensionless)	0.724	0.617	0.699	0.753	0.265	0.684	0.247	0.684 (0.258–0.736)	0.346 (0.229–0.569)	0.052
Age at diagnosis	2.5 wk	5 wk	7.3 y	61 y	2.5 wk	17 y	51 y			
Hormonal deficiencies	TSH, PRL	TSH, PRL	TSH, PRL^b^	TSH	TSH, T	TSH	TSH			
Replacement	LT4	LT4	LT4^a^	-	LT4, T	LT4	-			
Free T4 (pmol/L)^a^	15.3	18.4	11.3	7.3	17.5	10.6	8.8			
TSH (mU/L)^a^	0.01	<0.01	0.09	2.2	<0.01	0.79	0.58			
Prolactin (µg/L)^a^	3.5	<1	<1	6.3	12.5	8.5	5.5			
GH (µg/L)^a^	0.04	0.02	1.41	0.04	0.04	1.23	0.11			
Testicular volume (SDS)^c^	3.52	6.50	4.65	2.32	5.60	2.46	5.13			

Deconvolution of plasma GH secretion profiles in IGSF1-deficient male patients and controls. Basal, pulsatile, and total secretion of GH were log-transformed for normality. Data are presented as mean ± SD or median (P10-P90). *P* < 0.05 are marked in bold.

Abbreviations: ApEn, approximate entropy; LT4, levothyroxine; T, testosterone.

^a^Collected at 8:00 am (fasting), in-house reference ranges: free T4 12.0-22.0 pmol/L, TSH 0.300-4.800 mU/L, IGF-1 SD scores based on age-dependent in-house normal values, GH 0.00-1.31 µg/L, prolactin 4.0-15.0 µg/L, T 8.0-31.0 nmol/L, cortisol 0.100-0.600 µmol/L.

^b^Partial GH deficiency from age 7 to 17 years and treated with rhGH replacement in that period.

^c^Ultrasonographic volume of largest testis in SD scores (14).

To have a larger dataset with children and adults for analyses of head circumference and IGF-1, we combined data from the targeted cohort with available data from the large previously published cohort for these parameters. This enabled us to assess correlations between head circumference and thyroid function in 41 cases aged 0.4 to 89.5 years and demonstrate differences between childhood and adult IGF-1 standard deviation scores (SDS) in 55 cases aged 0.2 to 88 years. In addition, TRH tests with measurement of GH at baseline, and at 20 and 60 minutes after 200 μg of IV Protirelin (TRH, Alliance Pharmaceuticals Ltd, UK) performed in 8 members of the targeted cohort were supplemented with available data of 4 patients from the previously published cohort.

Clinical and biochemical measurements were compared with reference values either obtained from published literature or generated locally from control subjects. Assessment included anthropomorphology (18–20), endocrine biochemical evaluation using early morning samples, measured in locally available assays, ultrasonographically measured testicular size (21), and BMI- and height-corrected bioelectrical impedance analysis of body composition (22) using the Bodystat 1500MDD (Bodystat Limited, Douglas, United Kingdom). Radiography of the left hand was used to compute soft-tissue thickness (difference between second finger proximal phalanx and total finger diameter), as is standard practice in clinical medicine, irrespective of the handedness of the patient (23).

#### Evaluation of acromegalic facial characteristics.

Frontal and lateral photographs of the face were analyzed as previously described using the Facial Image Diagnostic Aid software tool, which detects features of acromegaly with higher accuracy than a medical expert (15–17). Control images were obtained from 18 nonacromegalic-, age-, sex-, and BMI-matched subjects (15–17). Images were individually classified by comparison with a training database comprising 64 subjects with confirmed acromegaly and 230 control subjects using a maximum likelihood classifier, assigning images to acromegalic or nonacromegalic categories when the respective probability was greater than 50.0%. The probability of the subject belonging to either group according to the presence of acromegalic features was noted.

#### 24-hour GH secretion patterns in patients.

In 7 male patients, GH was measured at 10-minute intervals for 24 hours as described previously (18). Secretion and elimination rates were calculated using a validated automated deconvolution method (2, 18–20) and the orderliness and consistency of subpatterns were assessed with the approximate entropy (21, 22). The outcome parameters of the deconvolution analysis were: pulse frequency (burst number/24 hour), half-life (minutes), secretion rates (µg/L/24 hour), and waveform shape (mode in minutes).

### Mice: general characteristics

*Igsf1* ^Δexon1^ and *Igsf1*^Δ312^ null mice were described previously. TSH synthesis is decreased in both genotypes of knock-out mice. T4, T3, and TSH are variably affected in *Igsf1*^∆exon1^ mice and equivalent to wild-type (WT) littermates in *Igsf1*^∆312^ mice, although TRH action is impaired in the pituitaries of both species (6, 23). Here, heterozygous *Igsf1*^Δexon1^ or *Igsf1*^Δ312^ females were crossed with C57BL6/J males (*Igsf1*^Δexon1^) or C57BL6/N males (*Igsf1*^Δ312^), and WT and IGSF1-deficient males were compared. Animals were housed in temperature- and light-controlled rooms (21-22°C, or 23°C, 12-hour light) with ad libitum access to food and water. No mice in this study were treated with synthetic thyroid hormone.

#### Murine auxology.

Mice were weighed weekly from birth and body composition evaluated by time-domain nuclear magnetic resonance using a minispec Live Mice Analyzer LF90 (Bruker). Following euthanasia by carbon dioxide asphyxiation at age 44 weeks, nose to tail base length was recorded using a digital Vernier caliper (accuracy ± 0.1 mm), liver and kidneys were measured using a digital balance accurate to the nearest 1 mg, and the skeleton was harvested and either stored directly in 70% ethanol or fixed in 10% neutral buffered formalin and subsequently stored in 70% ethanol.

### Murine skeletal analyses

#### Digital radiograph microradiography.

Long bones and tail vertebrae (Ca6, Ca7) were fixed in 70% ethanol. Soft tissue was then removed and digital radiograph images were recorded at 10 µm resolution using a Faxitron MX20 operating at 26 kV and 5x magnification (Qados, Cross Technologies plc, Sandhurst, Berkshire, UK). Bone length and relative bone mineral content were determined as previously described (24).

#### Micro-CT.

Femurs were analyzed by Micro-CT (Scanco uCT50, Scanco Medical, Switzerland; 70 kV, 200 mA, 0.5-mm aluminum filter) and cortical (tissue volume, bone volume, cortical thickness, bone mineral density, medullary and total diameters) and trabecular (bone volume/tissue volume, trabecular number, thickness, spacing) parameters determined. Cortical bone was analyzed at a 10-µm voxel resolution in a 1.5-mm region of interest (ROI) centered 56% of the length of the femur distal to the femoral head. Trabecular bone was analyzed at a 5-µm voxel resolution in a 1-mm long ROI beginning 100 µm proximal to the distal femoral growth plate. Scanco Medical 3D analysis software was used for all analyses and calculations (25). To generate high-resolution 3-dimensional images, 16-bit tiff images were imported into 32-bit Drishti (Australian National University Supercomputer Facility, http://anusf.anu.edu.au/Vizlab/drishti/) and ROIs were rendered using 64-bit Drishti.

#### Biomechanical testing.

Bones were stored and tested in 70% ethanol. Destructive 3-point bend testing of the femur and compression testing of caudal vertebrae 6 and 7 were performed using an Instron 5543 load frame and 100N and 500N load cells, respectively (Instron Limited, High Wycombe, Buckinghamshire, UK). Biomechanical variables of bone strength (yield load, maximum load, and stiffness) were determined from load-displacement curves as previously described (25, 26).

#### Skeletal preparations and histology.

Limbs were fixed in 10% neutral buffered formalin for 24h and decalcified in 10% EDTA for 21 days. Paraffin-embedded tibial sections were stained with van Gieson and Alcian blue and images of the proximal growth plates obtained. Growth plate dimensions were determined in ImageJ 1.41 software (http://rsb.info.nih.gov/ij/) with measurements in at least 4 separate positions. Mean values were calculated for the heights of the reserve zone, proliferative zone, hypertrophic zone, and total growth plate.

### Murine IGF-1 and GH pituitary content measurements

IGF-1 was measured using a rat/mouse IGF-1 2-site immunoenzymometric assay (IDS Diagnostic, Tyne and Wear, UK). Blood was obtained by tail-bleed from mice with mean age 10 weeks, the morning after an overnight fast, using sodium-heparinized tubes. Pituitary GH content was measured during the light phase by ELISA in *Igsf1*^Δ312^ mice aged 8 weeks and by radioimmunoassay in *Igsf*^*1*Δexon1^ mice as described previously (27, 28).

#### Gh pituitary mRNA measurements.

Total RNA was extracted from frozen, homogenized pituitary glands in TRIzol from *Igsf1*^Δ312^ mice aged 10 weeks. Following reverse transcription, quantitative PCR was performed using EvaGreen 2X quantitative PCR MasterMix-S (Applied Biological Materials Inc., Richmond, British Columbia, Canada), and *Gh* or *Rpl19* primers, running each sample in duplicate, and normalizing to *Rpl19* using the 2^-ΔΔCt^ method (29).

#### Murine GH secretion profiling.

Pulsatile GH secretion was measured in 6- and 9-week-old mice, as described by Steyn et al. (27) by collecting whole blood from the tail vein every 10 minutes for 6 consecutive hours beginning at 8:00 am. GH was measured by ELISA.

#### Murine GHRH test.

An IP GHRH injection (mGRF; 1 mg/kg; SCP0160, Sigma, Oakville, Canada) in saline was administered to mice aged 8 to 14 weeks, at 7:45 am with blood sampling for GH ELISA at baseline and 5, 10, 20, 40, and 60 minutes postinjection, as previously described by Steyn et al. (30).

#### Somatotrope quantitation.

Pituitaries were harvested from mice aged 6 to 7 weeks. All cells from individual dissected pituitaries were seeded on poly-D-lysine–coated coverslips, then fixed with 4% paraformaldehyde. Immunostaining was performed using a primary rabbit anti-rat GH antibody (1:500; AFP5641801, NIDDK) and a secondary goat anti-rabbit immunoglobulin IgG conjugated with Alexa fluor 488 (1/1000; Molecular Probes, Invitrogen). GH-positive cells were calculated as the ratio of GH stained cells over total number of cells per coverslip.

### Statistical comparison of group differences

Unless otherwise stated, group differences were compared using the 2-tailed independent Student *t* test or, in case the assumption of normality was not met (Shapiro-Wilk test), the Mann-Whitney *U* test. Categorical data were compared using the chi-square test (1-sample when compared with population data) or, when the expected count per cell was less than 5, Fisher’s exact test. Data were presented as mean ± SD or SEM, or, in case of non-normality, median (P10–P90). Correlations were assessed using the Pearson product-moment correlation coefficient or, for data that lacked a normal distribution, the Spearman rank correlation coefficient. Linear regression analysis was used to correct for age in the relation between years of untreated CCeH and BMI. Differences were considered statistically significant at *P* < 0.05. Growth curves were analyzed using a linear mixed-effects model of weight versus age with genotypes as fixed effects and subjects (mice) as random factors, with a 2-way ANOVA to test the overall effect of genotype on growth followed by Tukey’s post hoc tests using GNU licensed R software for statistical computing and graphics (31).

## Results

### Clinical indices of GH action in IGSF1-deficient male patients

End-organ sequelae of GH excess are typified by the clinical phenotypes associated with gigantism or acromegaly, in which excessive GH secretion occurs either before or after fusion of the epiphyses, respectively. Indices of GH excess and its associated sequelae were first assessed in the targeted cohort of 21 IGSF1-deficient males aged 19 to 89 years (Table 1). Digital analysis of acromegaloid facial features classified 52.4% of adult male patients as acromegalic compared with 19.1% of controls (*P *= 0.024). Acral growth analysis using left hand radiographs (data unavailable in 1 patient) showed left index finger soft-tissue thickness SDS to be elevated in IGSF1-deficient patients. There was no correlation between soft-tissue thickness SDS and either BMI (*r *= 0.280, *P *= 0.233), waist circumference (*r *= -0.141, *P *= 0.554), or fat percentage (*r *= -0.127, *P *= 0.592), suggesting this was not a consequence of obesity. Ten of 20 available hand radiographs also demonstrated features consistent with osteoarthritis, predominantly affecting the carpometacarpal and interphalangeal joints. Affected patients were 58 to 89 years old, whereas those without osteoarthritis were 19 to 55 years old.

Individuals in the targeted cohort were also evaluated for symptoms of GH excess using direct questioning. One patient experienced a change in shoe size (increase of 1 full UK shoe size between the ages of 22 and 73 years old), and no one in ring size. Symptoms consistent with sleep apnea were present in 3 patients (the diagnosis was confirmed in 1), occasional acroparesthesia in 3 patients and oily skin in 6 patients. No one reported malodorous sweating.

**Figure 1.**
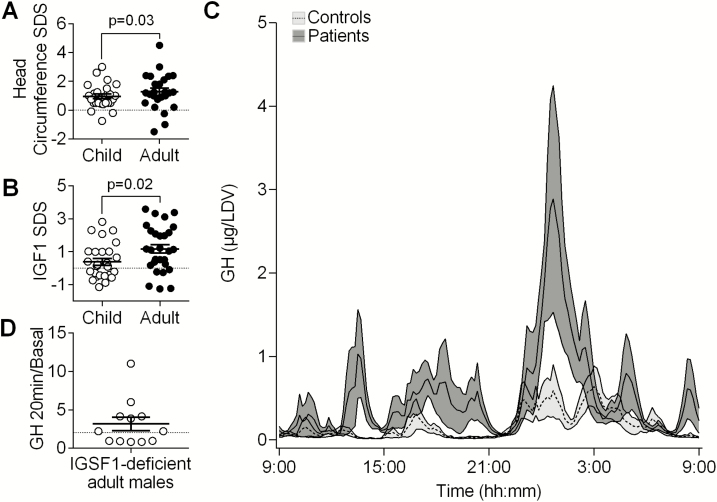
Measurements in IGSF1-deficient patients. (A) Head circumference SDS for 26 IGSF1-deficient children aged 1 to 16 years and 25 male adult patients aged 19 to 89.5 years. (B) IGF1 SDS in children aged 0.2 to 17.6 years (n = 27) and adults aged 18.1 to 88.1 years (n = 28). (C) 24-hour GH secretion profiles in micrograms per liter distribution volume for adult patients (n = 7, dark gray) and controls (n = 14, light gray), mean and SEM. (D) GH in micrograms per liter at 20 minutes/basal GH during a 200 µg TRH stimulation test in 12 adult patients, with values above the dotted line representing a paradoxical increase (peak to basal GH ratio > 2 µg/L). (A and B) Bars denote mean and SEM, and broken lines denote the 0 SD reference value. *P* values were calculated using a Mann-Whitney *U* test. Abbreviations: SDS, standard deviation score; TRH, thyrotropin-releasing hormone.

Head circumference SDS in the targeted cohort was found to be significantly increased (*P* < 0.001). Although all evaluated patients had CCeH, which may also cause macrocephaly, evaluation of head circumference in a larger group of IGSF1-deficient patients including available data from the previously published cohort (total n = 41, ages 0.4-89.5 years) confirmed that this did not correlate with free thyroxine levels at the time of evaluation (*P *= 0.8) or with age at commencement of levothyroxine (*P *= 0.4). In these 41 patients, head circumference SDS was most markedly elevated in adults (median [P10-P90]: 1.25 SDS [0.2-2.35 SDS]) versus children (0.5 SDS [-0.02 to 1.1 SDS], *P *= 0.03, Fig. 1A).

Adult height has previously been reported to be normal in IGSF1 deficiency (1), and patients in the targeted cohort also attained normal height, even when CCeH was first diagnosed in adulthood. No correlation was observed between years of untreated CCeH and adult height.

Four patients had been diagnosed with and treated for hypertension, all of whom were older than 60 years. In the remaining patients, median systolic and diastolic blood pressure were within the World Health Organization recommended target range (125 mm Hg [P10-P90: 110-135 mm Hg] and 75 mm Hg [P10-P90: 64-80 mm Hg], respectively). Two individuals had type 2 diabetes and 1 gave a history of prostate cancer and non-Hodgkin lymphoma. Lumbar bone mineral density was normal in 6 evaluated patients (mean, 1.14 ± 0.19 g/cm^2^; T-score, 0.3 ± 1.2 SD).

Bioelectrical impedance analysis of body composition was used to evaluate lean and fat mass proportions in the targeted cohort because we hypothesized that lean mass may be increased and fat mass decreased in the context of GH excess. Most patients were overweight (BMI > 25 kg/m^2^ in 76%, with 19% exhibiting severe obesity [BMI > 30 kg/m^2^]), compared with prevalences of 54% and 14%, respectively, in the Dutch population (*P* < 0.001 and *P *= 0.53, respectively) (32). However, contrary to our hypothesis, both the fat mass index (median, -0.08 SDS; interquartile ratio, -0.83 to 1.59 SDS; n = 19) and fat free mass index (0.40 SDS, -0.54 to 1.01 SDS, n = 16) were comparable to that observed in a BMI-matched reference population (*P *= 0.175 and *P *= 0.8, respectively). Years of untreated CCeH, corrected for age, did not correlate with BMI of body composition.

### Biochemical assessment of GH secretion in IGSF1-deficient patients

Having established that IGSF1-deficient men exhibit acromegaloid characteristics, we next evaluated biochemical parameters of GH secretion. Median serum IGF-1 SDS was significantly increased in the targeted cohort (Table 1), and analysis of a larger group including available data from our previously published cohort (total n = 55) demonstrated that the increased IGF-1 SDS was most marked in adults (Fig. 1B), as previously reported (4).

We then delineated GH load directly by assessing temporal GH secretion in 7 IGSF1-deficient adult males with comparable endocrine and phenotypic characteristics to the rest of the targeted cohort, and 14 age-, sex-, and BMI-matched controls (Table 2). Median basal, pulsatile, and total GH secretion were significantly elevated and exhibited a tendency to reduced secretory regularity as evidenced by increased approximate entropy in 5 of the 7 subjects (*P *= 0.05) (Fig. 1C, Table 2). No differences were observed in pulse frequency, waveform shape, half-life, or Weibull gamma distribution (a marker of pulse regularity). There was no correlation of higher basal, pulsatile, or total GH secretion with adult height, lean body mass, or finger soft tissue thickness (data not shown).

We also evaluated GH increment following peripheral TRH injection in 12 patients. This test has been used in the diagnosis of acromegaly, where a paradoxical response, defined as peak to basal GH ratio > 2 during a standard 200 µg TRH test, is observed in 50% to 75% cases (33). Seven of 12 evaluated adult IGSF1-deficient patients exhibited a paradoxical GH increase (Fig. 1D).

### GH biochemistry in IGSF1-deficient mice

**Figure 2.**
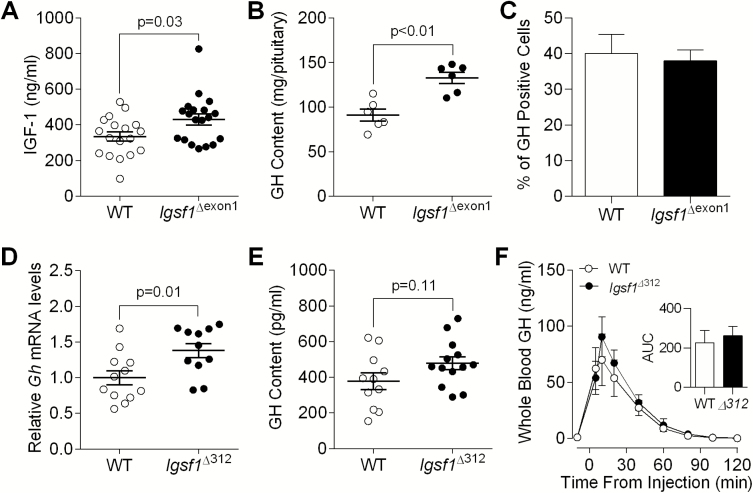
(A) Plasma IGF-1 in 19 *Igsf1*^∆exon1^ and 18 WT mice, mean age 10 weeks (range, 8-11.9 weeks *Igsf1*^∆exon1^ and WT mice). (B) Pituitary GH protein content in adult male WT and *Igsf1*^∆exon1^ mice aged 8 weeks (n = 6 each). (C) GH-positive cells (GH stained cells/total number of cells) from WT (n = 4) or *Igsf1*^∆exon1^ mice (n = 3 age 6-7 weeks). (D) Relative pituitary *Gh* mRNA and (E) protein content in WT and *Igsf1*^∆312^ mice aged 10 and 8 weeks of age, respectively (n = 11-13). (F) Mean GH response to GHRH in n = 12 WT and n = 16 *Igsf1*^∆312^ mice aged 8 to 14 weeks. Inset illustrates the area under the curve. Data presented as mean and SEM. *P* values were calculated using a Mann-Whitney *U* test or an unpaired, 2-tailed student *t* test. Abbreviation: WT, wild-type.

We next evaluated biochemical indices of GH production in male *Igsf1*^∆exon1^ mice to investigate whether somatotrope function in this model recapitulated the findings in IGSF1-deficient humans. Six-hour GH secretion profiles in 6- and 9-week-old *Igsf1*^∆exon1^ and WT males were not significantly different (Table 3). However, median serum IGF-1 was significantly elevated in 10-week-old *Igsf1*^∆exon1^ mice compared with WT animals (441 [P10-P90: 286-541] vs. 345 [P10-P90: 220-463] ng/mL, *P *= 0.03) (Fig. 2A), consistent with excess GH secretion.

**Table 3.** Deconvolution of Plasma GH Blood Profiles in WT and *Igsf1*^∆312^ Mice

	Mice				P Value^a^		
	WT Aged 6 Wk	WT Aged 9 Wk	*Igsf1* ^∆312^ Aged 6 Wk	*Igsf1* ^∆312^ Aged 9 Wk	Between Groups	Time	Time × Group
Pulse frequency (no./6 h)	4.6 ± 0.4	4.3 ± 0.3	4.7 ± 0.5	3.6 ± 0.4	0.58	0.048	0.23
Mode (min)	15.3 ± 1.0	16.3 ± 0.6	14.8 ± 0.9	15.2 ± 0.8	0.30	0.46	0.76
Slow half-life (min)	6.4 ± 0.6	6.2 ± 0.7	7.2 ± 0.6	7.1 ± 0.6	0.18	0.81	0.99
Basal secretion (ng/mL/6 h)	222 ± 127	313 ± 104	110 ± 28	171 ± 28	0.72	0.029	0.86
Pulsatile secretion (ng/mL/6 h)	539 ± 118	721 ± 148	801 ± 166	652 ± 140	0.52	0.96	0.27
Total secretion (ng/mL/6 h)	762 ± 226	1034 ± 219	912 ± 185	823 ± 157	0.62	0.62	0.32
Mean pulse mass (ng/mL)	132 ± 33	179 ± 35	186 ± 36	235 ± 60	0.37	0.33	0.68
ApEn (dimensionless)	0.791 ± 0.067	0.748 ± 0.069	0.804 ± 0.074	0.649 ± 0.067	0.61	0.056	0.28

Deconvolution analysis of plasma GH secretion profiles in male *Igsf1*^Δexon1^ and WT mice. Calculations were performed with an automated Matlab deconvolution program (34). Data are shown as mean ± SEM.

Abbreviations: ApEn, approximate entropy; WT, wild-type.

^a^Data were analyzed with the 2-way ANOVA for repeated measures, assessing differences between WT and *Igsf1*^*Δ312*^ mice (between groups), differences between age 6 and 9 weeks (time), and the interaction between the effects of group and time (time × group). Where required, data were logarithmically transformed. The data at 6 and 9 weeks are from the same animals.

We also used this model to further delineate pituitary GH synthesis. Pituitary GH protein levels were elevated in 8-week-old *Igsf1*^∆exon1^ mice, although *Gh* mRNA levels were previously shown to be normal in this strain (Fig. 2B, (1)). Somatotrope numbers were comparable in *Igsf1*^∆exon1^ mice and WT controls aged 6 to 7 weeks (Fig. 2C). In contrast, in a second IGSF1-deficient model, *Igsf1*^∆312^ (6), pituitary *Gh* mRNA levels were elevated in 10-week-old mice but GH protein content was the same in 8-week-old WT and *Igsf1*^∆312^ mice (Fig. 2D, E).

A GHRH stimulation test showed a tendency to a larger GH response in *Igsf1*^∆312^ mice aged 8 to 14 weeks compared with WT controls, but responses in both groups were variable and the difference was not statistically significant, as indicated by the area under the curve (Fig. 2F and inset).

### Indices of growth hormone action in *Igsf1*^∆exon1^ male mice

**Figure 3.**
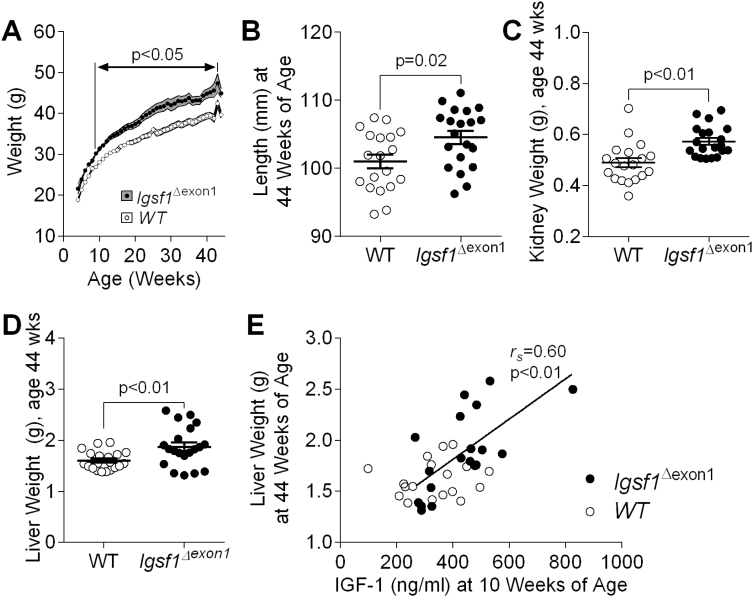
Male *Igsf1*^∆exon1^ and WT mouse measurements at 44 weeks (n = 19-20 mice per genotype). (A) Longitudinal body weights (n = 8-20 mice per genotype). (B) Body length, and (C) kidney and (D) liver weights at 44 weeks of age. (E) Correlation between liver weight at 44 weeks of age and plasma IGF-1 levels in *Igsf1*^∆exon1^ (n = 19) and WT (n = 18) mice aged 10 weeks. Line depicting the significant correlation in *Igsf1*^∆exon1^ mice. (B-D) Data presented as mean and SEM. *P* values were calculated using a Mann-Whitney *U* test or an unpaired, 2-tailed Student *t* test. (E) *P* values were calculated using Spearman correlation analysis. Black circles: *Igsf1*^∆exon1^ mice; white circles: WT mice. Abbreviation: WT, wild-type.

We next interrogated an acromegaloid phenotype in male *Igsf1*^∆exon1^ mice as a consequence of increased IGF-1 action. Comparison of *Igsf1*^∆exon1^ with WT males demonstrated a sustained 11% to 13% increase in body weight, which achieved significance by age 10 weeks (*P *= 0.035), persisted at 44 weeks *(P* < 0.01) (Fig. 3A), and was associated with increased mean body length and kidney and liver weights at 44 weeks of age (Fig. 3B–D). Increased circulating IGF-1 levels from *Igsf1*^∆exon1^ mice at 10 weeks of age correlated with increased hepatic weight of these animals at 44 weeks of age, while no correlation was observed in WT animals (Spearman rho = 0.6, *P *= 0.006, Fig. 3E).

**Figure 4.**
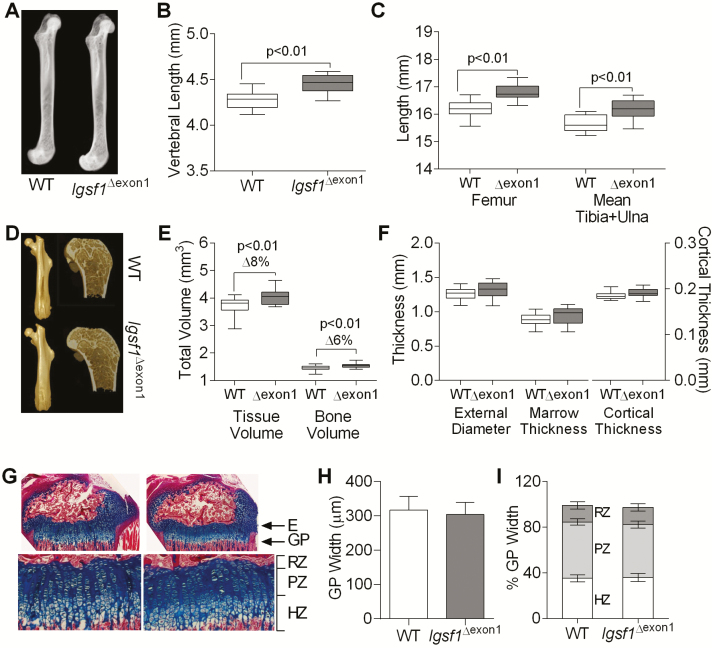
Skeletal analyses in mice. (A-C) Faxitron X-ray skeletal microradiography and (D-F) microcomputed tomography analysis of skeletal architecture in male *Igsf1*^∆exon1^ and WT mice at 44 weeks of age (n = 19-20). (A) Representative images of femurs (Faxitron X-ray) from *Igsf1*^∆exon1^ and WT mice. (B) Vertebral length, and (C) femoral and combined tibia and ulnar length were increased in *Igsf1*^∆exon1^ mice. (D) Representative images of femur (microcomputed tomography) from WT (top) and *Igsf1*^∆exon1^ (bottom) mice. (E) Femoral tissue volume and bone volume were increased in *Igsf1*^∆exon1^ mice. (F) External and internal femoral diameters and cortical thickness. (G) Proximal tibia growth plates from 10 WT and 6 *Igsf1*^∆exon1^ mice, mean age P20.5, stained with Alcian blue (cartilage) and van Gieson (osteoid) with graphs (H, I) showing mean dimensions. Abbreviations: E, epiphysis; GP, growth plate; H, hypertrophic zone; PZ, proliferative zone; RZ, reserve zone; WT, wild-type. Error bars represent SD. Box-whisker plots: boxes extend from 25th to 75th percentiles, the horizontal line represents the median and the vertical bars the minimum and maximum values. Data presented as mean and SEM, *P* values were calculated using an unpaired, 2-tailed student *t* test.

*Igsf1* ^∆exon1^ mice also exhibited globally increased skeletal dimensions at 44 weeks of age (Fig. 4A–E and Table 4). Long bone architecture assessed by Faxitron X-ray skeletal microradiography (Fig. 4A) demonstrated a proportionate increase in bone length in *Igsf1*^∆exon1^ mice (Fig. 4B, C). Using high-resolution microcomputed tomography (Fig. 4D), we observed an increase in total tissue and bone volume (Fig. 4E), with a tendency for increased cortical thickness (Fig. 4F, Table 4), with comparable trabecular parameters and bone mineral density to controls (Table 4). Bones from *Igsf1*^∆exon1^ mice were stronger, with higher yield load noted for both femur and vertebrae, and higher vertebral maximum load, as predicted for normally mineralized bones with proportionately larger internal and external diameters (Table 4). Despite the differences in final bone length, no difference in growth plate morphology was observed at P20.5 (Fig. 4G–I).

**Table 4.** Auxological Parameters in Wild-Type and *Igsf1*^∆exon1^ Male Mice Aged 44 Weeks

Parameter	*Igsf1* ^∆exon1^	Wild-type	P Value
Combined tibia and ulnar length^a^ (mm)	16.2 (15.8–16.6)	15.6 (15.4–16.0)	<0.0001
Femur length (mm)	16.8 ± 0.06	16.2 ± 0.06	<0.0001
Vertebral length (mm)	4.46 ± 0.02	4.28 ± 0.02	<0.0001
Femoral tissue volume^a^ (mm^3^)	4.06 (3.74–4.27)	3.81 (3.52–4.04)	0.002
Femoral bone volume (mm^3^)	1.54 ± 0.02	1.46 ± 0.02	0.004
Femoral bone volume/tissue volume	0.38 ± 0.005	0.39 ± 0.005	0.2
Femoral cortical thickness^a^ (mm)	0.189 (0.179–0.200)	0.183 (0.177–0.196)	0.05
Bone mineral density^a^ (mgHA/cm^3^)	1007 (994–1069)	1012 (969–1060)	0.6
Number of trabeculae, distal tibia	3.45 ± 0.08	3.45 ± 0.07	1.0
Trabecular thickness, distal tibia (mm)	0.058 ± 0.001	0.057 ± 0.001	0.7
Femoral yield load (N)	14.22 ± 0.32	12.67 ± 0.32	0.002
Vertebral yield load^a^ (N)	114.8 (105.1–134.7)	103.1 (88.7–115.1)	0.004
Femoral maximum load (N)	21.0 ± 0.5	20.3 ± 0.6	0.4
Vertebral maximum load (N)	135.5 ± 2.59	120.3 ± 2.46	0.0001
Femoral stiffness (N/mm)	127.6 ± 3.74	128.8 ± 3.16	0.8
Vertebral stiffness (N/mm)	452.8 ± 9.36	428 ± 11.3	0.1
Mean body length (mm)	104.6 ± 0.97	101.0 ± 1	0.02
Liver weight (g)	1.87 ± 0.09	1.60 ± 0.04	<0.01
Kidney weight^a^ (g)	0.55 (0.51–0.67)	0.48 (0.42–0.57)	<0.001
Lean mass (g) 10 wk	23.1 ± 0.37	20.7 ± 0.38	<0.0001
Fat mass (g) 10 wk	2.59 ± 0.18	2.29 ± 0.13	0.2
Lean mass (g) 44 wk	27.8 ± 0.44	24.6 ± 0.37	<0.0001
Fat mass (g) 44 wk	11.7 ± 0.85	9.8 ± 0.70	0.09
Percentage fat mass 44 wk	25.3 ± 1.27	24.3 ± 1.42	0.6
Percentage lean mass 44 wk	61.8 ± 1.13	62.4 ± 1.25	0.7

Auxological parameters in male *Igsf1*^Δexon1^ and wild-type (WT) mice aged 44 weeks with values from 19–20 mice of each genotype. Values represent mean ± SEM with *P* values calculated using an unpaired, 2-tailed student *t* test or, ^a^for parameters where either genotype was not normally distributed, median (P10-P90) with *P* values calculated using a Mann-Whitney *U* test.

**Figure 5.**
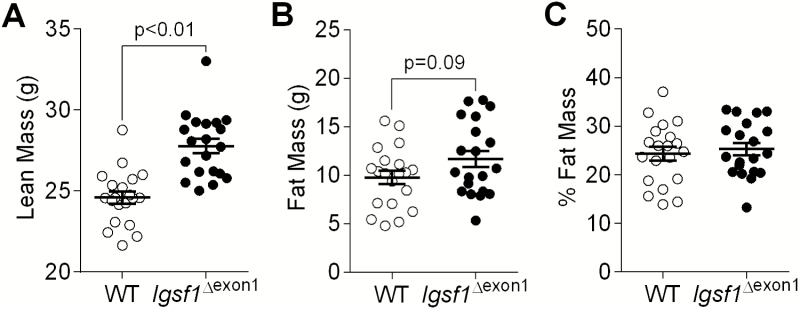
Body composition of male *Igsf1*^∆exon1^ and WT mice at 44 weeks of age (n = 19-20 mice per genotype). (A) Lean mass in grams. (B) Fat mass in grams. (C) Percentage fat mass. Data presented as mean and SEM, *P* values were calculated using an unpaired, 2-tailed student *t* test.

Lean body mass was also significantly increased in *Igsf1*^∆exon1^ mice aged 10 weeks and 44 weeks (Fig. 5A, Table 4); however, there was also a tendency for increased fat mass, which did not reach significance (Fig. 5B). As such, percentage fat and fat-free masses were not different between genotypes (Fig. 5C, Table 4).

## Discussion

This is the first study to evaluate the role of IGSF1 in human or murine somatotrope function, and our findings are consistent with end-organ sequelae of GH excess in both species. GH hypersecretion occurring after epiphyseal fusion may result in a constellation of clinical features typically seen in acromegaly, including altered facial appearance, characterized by prominence of the supraorbital ridge, broad nose, large lips, prognathism, macroglossia, and coarsening of the facial features. Bony expansion and soft-tissue swelling may cause acral enlargement, and patients may report oily skin and hyperhidrosis. Systemic consequences of increased GH levels include widespread complications such as sleep apnea and ventilatory dysfunction, insulin resistance and dyslipidemia, hypertension, ventricular hypertrophy and cardiomyopathy, as well as early-onset osteoarthritis. The risk of certain malignancies, especially colon and thyroid, may also be increased (35, 36). If the onset of hypersomatotropism precedes long bone epiphyseal fusion, the predominant effect of GH excess is to promote linear growth, resulting in increased height (37).

The most frequent manifestations of acromegalic GH excess include characteristic facies and increased acral growth, both of which were detected in our IGSF1-deficient patient cohort (35). The majority of patients were classified as acromegalic following digital analysis of facial photographs, substantiating previous anecdotal reports of acromegalic facial features in patients harboring *IGSF1* mutations. Head circumference SDS was increased and did not correlate with thyroid status. Excessive acral growth was evidenced by elevated finger soft-tissue thickness SDS. Evaluation of *Igsf1*^∆exon1^ mice revealed globally increased organ size and skeletal dimensions, also consistent with GH-mediated effects.

Adult height was not increased in patients in this study, and IGSF1 deficiency has previously been associated with normal adult stature (1, 3). The multiple childhood endocrinopathies in these patients may have complex influences on linear growth, with CCeH and transient GH deficiency predicted to have a negative impact, whereas pubertal delay may predispose to greater adult height (38, 39). Additionally, the timing of GH hyperstimulation in IGSF1 deficiency may be crucial for determining the consequent end-organ sequelae. The age of onset of GH hypersecretion was not defined in this study; however, the more markedly elevated IGF-1 levels and elevated head circumference SDS in adults compared with children and apparently paradoxical occurrence of childhood GH deficiency in a minority of cases would support a later onset GH hypersecretion. A gradually progressive adolescent or adult-onset GH excess may also explain why patients exhibit acromegalic characteristics rather than increased height because acromegalic features develop as a result of bone expansion after epiphyseal fusion has occurred without increased linear growth.

In contrast, *Igsf1*^∆exon1^ mice exhibit a global increase in body size and skeletal dimensions. This is highly likely to be driven by excess GH and is particularly notable in the context of CCeH, which usually results in growth retardation from the critical regulation of linear growth by thyroid hormones. Unlike humans, *Igsf1*^∆exon1^ mice exhibit increased body size and lean mass at 10 weeks of age, suggesting that GH production is already elevated during the pubertal transition, before adulthood. Because murine long bone epiphyses do not fuse, permitting continued slow, longitudinal growth into adulthood such continued growth, as well as an earlier onset GH excess may also explain the increase in murine skeletal dimensions, which is not observed in humans (40).

Although GH excess generally promotes increased lean and decreased fat mass, IGSF1-deficient patients exhibited increased BMI with appropriate body composition for an overweight population, without observed increases in lean mass. These observations are consistent with the likely complex effects of IGSF1 deficiency on body composition, to which increased GH action is but one contributing factor. In particular, hypothyroidism and delayed puberty associated with IGSF1 loss of function may predominantly increase fat mass proportions, thereby negating some of the effects of GH excess on body composition (41). Moreover, it is possible that bioelectrical impedance analysis of body composition is not sensitive enough for measuring minor changes in lean body mass (42). In contrast, increased lean mass was detected in *Igsf1*^∆exon1^ mice, as expected in the context of their elevated IGF-1 levels. The reason for this difference between mice and humans was not explored; however, in contrast to humans, the murine gonadal axis seems relatively unaffected by IGSF1 deficiency, which may facilitate murine lean mass expansion in response to GH excess (23). There was a concomitant tendency to increased fat mass in *Igsf1*^∆exon1^ mice, which may have been due to untreated CCeH, or alternatively, a consequence of the globally increased body dimensions.

GH excess may also influence levels of thyroid hormone metabolites. Individuals harboring the p.L773P IGSF1 mutation have previously been noted to exhibit more markedly subnormal FT4 levels than FT3 levels, and decreased reverse T3 *z* scores (43). This biochemical pattern would be consistent with preferential deiodination of FT4 to FT3 at the expense of reverse T3 in order to mitigate tissue hypothyroidism. Differential, tissue-dependent regulation of deiodinase activity by GH has been proposed to produce a similar thyroid hormone signature, raising the possibility that peripheral effects of GH action also modulate thyroid hormone metabolism in the IGSF1 deficiency syndrome (44).

IGSF1-deficient patients in this study demonstrated clear biochemical evidence for increased GH secretion during 24-hour pulsatility studies, with increased basal, median, and pulsatile GH secretion. Additionally, median serum IGF-1 levels were increased. In *Igsf1*^∆exon1^ mice, 6-hour GH secretion profiles were not significantly different from WT animals. However, interindividual variability was high and the highly dynamic nature of GH production may have precluded detection of modest differences over this relatively short sampling period. Such variability has also been described in the context of central hypothyroidism in this mouse line, where evaluation of some cohorts of *Igsf1*^∆exon1^ mice have shown decreased levels of serum T3 or T4, but in other cohorts, hormone levels have been comparable with WT measurements (1, 45). Indirect evidence for increased GH production in both *Igsf1*^∆exon1^ and *Igsf1*^∆312^ mice includes the detection of elevated pituitary GH levels in *Igsf1*^∆exon1^ mice and elevated pituitary *Gh* mRNA levels in *Igsf1*^∆312^. Metabolically divergent background strains for these models (C57BL/6J for *Igsf1*^∆exon1^ and C57BL/6N for *Igsf1*^∆312^ mice) may have contributed to the discordant mRNA and protein results (46).

Ascertainment of the underlying mechanism for the observed somatotrope neurosecretory hyperfunction was beyond the scope of this study. Because IGSF1 is expressed in rat hypothalamus, including ~19% of *Ghrh*-expressing neurons, as well as in pituitary somatotropes, it may be implicated either in hypothalamic regulation of GH or play a somatotrope-specific role in GH production or secretion (1, 3). Autonomous GH secretion is unlikely because patients exhibited normal Weibull gamma measurement and only borderline elevated approximate entropy. Moreover, although GH secretion is strikingly increased in humans, the 2- to 3-fold increase is distinct from acromegalic values, in which increases of up to 50-fold are observed (47). In *Igsf1*^∆exon1^ mice, IGF-1 and size increments were comparably modest and somatotrope numbers were normal.

It has previously been proposed that IGSF1 has a modulatory effect on pituitary hormone synthesis rather than being an essential component of hormone synthesis pathways (45). Such a function would be consistent with the marked intra- and interfamilial phenotypic variability observed in humans harboring the same *IGSF1* loss-of-function mutations and also within and between litters born to congenic *Igsf1* knockout mouse strains (45). A recent study suggested that IGSF1 may inhibit TGFβ1 signaling, perhaps by interacting with the TGFβ type I receptor, ALK5, thereby attenuating TGFβ1’s suppression of *Trhr1* expression in the basal state. Although further validation is required to substantiate this proposed model, these observations support a candidate modulatory mechanism for central hypothyroidism in IGSF1 deficiency (48).

By analogy with the effects of IGSF1 on thyrotrope function, a modulatory influence on GH production rather than an obligatory role in GH synthesis seems plausible. However, difficulties in proposing a mechanism for the other axes of anterior pituitary dysfunction in IGSF1 deficiency include the lack of information regarding the IGSF1 interactome, the protein’s lack of defined functional domains, and that its normal function remains unclear (45). GH pulsatile secretion is subject to a complex interplay between stimulatory and inhibitory influences. Hypothalamic GHRH exerts the main feedforward effect and hypothalamic somatostatin, as well as circulating GH and IGF-1, mediate feedback inhibition. Additionally, centrally acting neuromodulators may influence activity of somatostatin and GHRH-expressing neurons (49). Either decreased feedback inhibition or increased GHRH secretion or sensitivity could explain our observations in IGSF1 deficiency. Future studies could include challenge with somatostatin analogues or IGF-1 to investigate the integrity of the pathways mediating GH inhibition, and human GHRH challenge to investigate whether the tendency to increased response in the mice is recapitulated in human patients. The observation that GH deficiency may occur in childhood, IGSF1 deficiency remains a conundrum in the context of the adult GH hypersecretion delineated in this study.

Although IGSF1 deficiency is associated with decreased TRH action in thyrotropes, the presence of a paradoxical GH response to TRH in patients does suggest that TRHR signaling is not globally decreased. However, the physiological significance of this observation with regard to the mechanism of GH hypersecretion in humans is unclear. TRH is known to have age- and endocrine environment-dependent effects on GH release in rats (50–52), but whether this translates to the human phenotype is unknown. A paradoxical GH response to TRH may also be observed in nonacromegalic conditions (eg, depression, anorexia nervosa, untreated primary congenital hypothyroidism) where it has been hypothesized to reflect dysregulation of the normal dopaminergic or somatostatin-mediated inhibitory effects on GH release, supporting the argument that although a somatotrope-autonomous role for IGSF1 is possible, additional hypothalamic dysfunction may also contribute to GH excess (53, 54).

The congenital nature of IGSF1 deficiency suggests that GH hypersecretion in affected patients is likely to persist lifelong, even if the onset occurs in adulthood. Therefore, although the 2- to 3-fold increase in GH secretion is significantly less than that seen in acromegaly, the chronicity of this effect may be more marked than in patients with acquired somatotrope hyperfunction. Larger clinical studies are needed to determine the long-term consequences of this moderate, chronic GH excess. Metabolic parameters including lipid concentrations, fasting glucose, and insulin have previously been shown to be normal in a larger cohort of adult males with IGSF1 deficiency. However, C-peptide was increased in 40% of previously evaluated males; therefore, future studies warrant oral glucose tolerance testing, both to investigate glucose and insulin responses as well as the extent of GH suppression in response to a standard glucose load (3). Hand radiographs demonstrated osteoarthritic features in 50% patients in the current study but the incidence of large joint or axial arthritis was not evaluated, and echocardiography was not performed to evaluate GH-induced cardiac consequences. Additionally, the age range and relatively small numbers of patients in our study do not enable us to comment on the risk of malignancy in IGSF1-deficient patients. Also, the magnitude of secreted GH levels and associated end-organ effects require evaluation in heterozygous female IGSF1 mutation carriers who also exhibit elevated mean IGF-1 levels, and are less likely than their hemizygous male counterparts to exhibit CCeH with its potentially mitigating effect on GH secretion (3).

Our observations identify a novel role for IGSF1 in GH physiology and extend the phenotype of the IGSF1 deficiency syndrome to include GH hypersecretion in affected adults with associated end-organ sequelae. A similar phenotype was observed in *Igsf1*-deficient mice, although further studies are required to delineate the underlying mechanism for these effects. Given the significant morbidity that may be associated with chronic GH excess, we suggest that clinical management of IGSF1-deficient patients should include vigilance for these long-term sequelae, especially in older adults.

## Abbreviations

BMI: body mass index
CCeH: congenital central hypothyroidism
CTD: carboxy-terminal domain
ROI: region of interest
SDS: standard deviation score
Trhr1: thyrotropin-releasing hormone receptor 1
TRH: thyrotropin-releasing hormone
WT: wild-type.

## Additional Information

***Disclosure summary***: The authors have nothing to disclose

***Data Availability:*** The datasets generated during and/or analyzed during the current study are not publicly available but are available from the corresponding author on reasonable request.
